# Less extreme and earlier outbursts of ice-dammed lakes since 1900

**DOI:** 10.1038/s41586-022-05642-9

**Published:** 2023-02-15

**Authors:** Georg Veh, Natalie Lützow, Jenny Tamm, Lisa V. Luna, Romain Hugonnet, Kristin Vogel, Marten Geertsema, John J. Clague, Oliver Korup

**Affiliations:** 1 Institute of Environmental Science and Geography, University of Potsdam, Potsdam-Golm, Germany; 2 Institute of Geosciences, University of Potsdam, Potsdam-Golm, Germany; 3 Potsdam Institute for Climate Impact Research, Potsdam-Telegrafenberg, Germany; 4 LEGOS, Université de Toulouse, CNES, CNRS, IRD, UPS, Toulouse, France; 5 Laboratory of Hydraulics, Hydrology and Glaciology (VAW), ETH Zürich, Zürich, Switzerland; 6 Swiss Federal Institute for Forest, Snow and Landscape Research (WSL), Birmensdorf, Switzerland; 7 Federal Institute for Materials Research and Testing (BAM), Berlin, Germany; 8 Ministry of Forests, Prince George, British Columbia Canada; 9 Department of Earth Sciences, Simon Fraser University, Burnaby, British Columbia Canada

**Keywords:** Natural hazards, Environmental impact, Hydrology

## Abstract

Episodic failures of ice-dammed lakes have produced some of the largest floods in history, with disastrous consequences for communities in high mountains^1–7^. Yet, estimating changes in the activity of ice-dam failures through time remains controversial because of inconsistent regional flood databases. Here, by collating 1,569 ice-dam failures in six major mountain regions, we systematically assess trends in peak discharge, volume, annual timing and source elevation between 1900 and 2021. We show that extreme peak flows and volumes (10 per cent highest) have declined by about an order of magnitude over this period in five of the six regions, whereas median flood discharges have fallen less or have remained unchanged. Ice-dam floods worldwide today originate at higher elevations and happen about six weeks earlier in the year than in 1900. Individual ice-dammed lakes with repeated outbursts show similar negative trends in magnitude and earlier occurrence, although with only moderate correlation to glacier thinning^8^. We anticipate that ice dams will continue to fail in the near future, even as glaciers thin and recede. Yet widespread deglaciation, projected for nearly all regions by the end of the twenty-first century^9^, may bring most outburst activity to a halt.

## Abstract (web summary)

An assessment of ice-dam failures in six mountain regions shows that extreme peak flows and volumes have declined sharply since 1900, and that ice-dam floods today originate at higher elevations and earlier in the year.

## Main

One of the major hazards caused by ongoing glacier retreat are sudden floods resulting from the failure of unstable glacier dams^4–7^. These ice dams temporarily and periodically impound rainfall and meltwater that can be released abruptly following dam flotation, subglacial tunnel enlargement or mechanical collapse^10^. Such glacier lake outburst floods (GLOFs) initiate hazard cascades with numerous impacts downstream. Historic ice-dammed GLOFs have travelled hundreds of kilometres, entraining sediment along their runout paths; widening river channels by bank erosion; triggering landslides from undercut and destabilized hillslopes; and covering valley floors with metre-thick sheets of sediment, ice and woody debris^11–14^. Extreme GLOF discharges can raise local river levels by several metres, increasing bed shear stresses and stream power values sufficiently to incise bedrock^15^. Floods from the largest lake outbursts can last for several days and destroy local flood control systems^7,16^. In many mountain regions, settlements, tourism, forestry and mining advanced into high mountain valleys during the twentieth century^17^ and thus closer to the source areas of GLOFs. As a consequence, ice-dam failures have caused many hundreds of fatalities, damaged infrastructure and farmland worth hundreds of millions of dollars, and disrupted transportation and communication routes^1^.

Despite the hazard posed by ice-dammed lakes, temporal trends in the magnitude and timing of their failures remain poorly understood in most mountain regions. We attribute the limited knowledge of contemporary GLOF activity to the largely unsystematic and regionally inconsistent record of GLOFs. How strongly, or whether at all, regional GLOF activity responds to accelerating glacier melt therefore remains an open question. Melting glacier margins create new space for ice-dammed lakes to form and grow^13,14,18^. Thus, in the future, ice dams might store an increasing amount of meltwater, causing larger floods if they fail. However, the few detailed case studies of ice-dammed lakes, particularly in Iceland and northwest North America^6,11,14,19^, suggest the opposite. There, individual ice-dammed lakes fill and empty repeatedly, releasing gradually smaller flood volumes over time. The edges of glacier dams might begin to float on the adjacent lake once a critical lake level is reached, suggesting that a gradually thinning glacier dam requires a lower threshold to initiate subglacial drainage^6,20,21^. Within this cycle, the timing of GLOFs, that is, the day of the year when a lake begins to drain, is likely to shift, given that thinner dams may require a shorter period of time to refill to their maximum storage capacity^14,22^. This cycle of GLOFs from a given lake may end when the ice dam has decayed to the point where it can no longer impound water^20,21^. Yet, new cycles of GLOFs might originate from higher elevations with ongoing exposure of ice-free areas at glacier margins^23^.

## A database of historic ice-dam failures

**Fig. 1:**
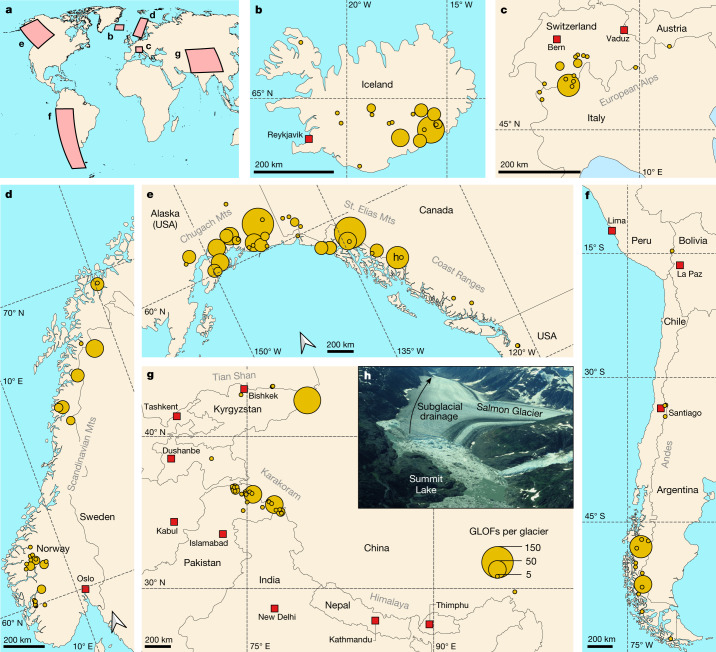
Map of dated GLOF source locations in the six study regions. **a**, Global overview. **b**, Iceland. **c**, European Alps. **d**, Scandinavia. **e**, Northwest North America. **f**, Andes. **g**, High Mountain Asia, including legend. We created all maps in **a**–**g** using the free software QGIS V3.22.6 (https://www.qgis.org/). Generalized country boundaries and locations of cities are available through the online ArcGIS hub hosted by Esri Data and Maps (https://hub.arcgis.com/). **h**, Typical setting of an ice-dammed lake: Summit Lake dammed by Salmon Glacier, British Columbia, Canada (see location in **e**). Oblique view shows the situation on 11 August 1989, when the lake was approximately 1-km wide. Summit Lake has been draining beneath Salmon Glacier (arrow) since 1961, and only a small lake exists today (Fig. 5c). Photograph by John J. Clague. Mts, Mountains.

**Extended Data Fig. 1:**
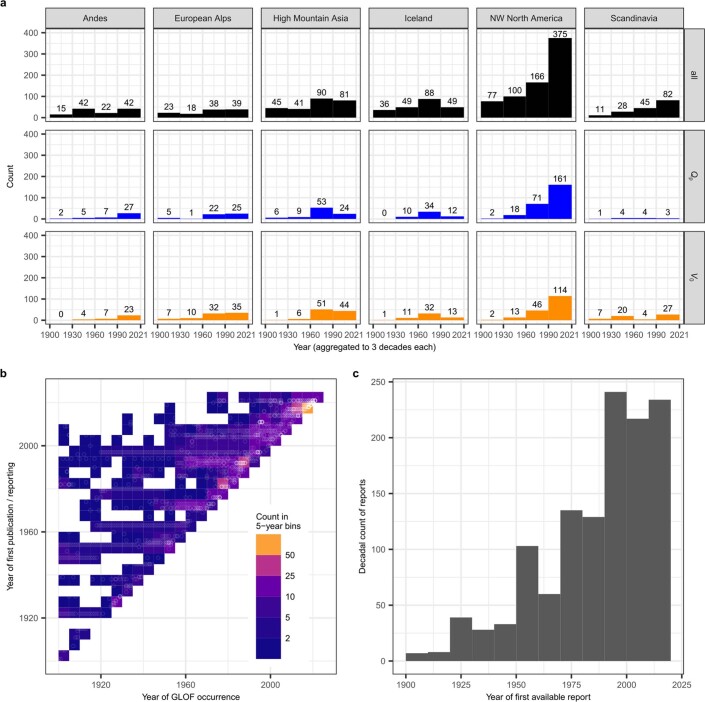
GLOF reporting between 1900 and 2021. **a**, Histograms of all reported GLOFs (top), reported GLOFs with documented peak discharge *Q*_p_ (middle), and reported GLOFs with documented flood volume *V*_0_. Numbers are the total number of GLOFs every three decades. **b**, Plot of year of GLOF occurrence versus year of first available document. White circles are individual GLOFs; colours are aggregated cases in 5-year intervals. **c**, Histogram showing the year of first available documentation for all cases in our database.

Here we study the core components of the GLOF cycle by examining historical changes in GLOF magnitude, timing and source elevation, and their relationships to glacier thinning. We focus on the six most glaciated mountain regions on Earth, that is, northwest North America, High Mountain Asia, the Andes, Iceland, Scandinavia and the European Alps (Fig. 1). In reviewing 446 sources of information, we found a total of 1,569 dated outbursts from 186 ice-dammed lakes in the period 1900–2021 (Methods). In 64% of all cases, we had access to primary sources such as original research papers, official documentation from local agencies, reports in newspapers and social media platforms, and written correspondence with local eyewitnesses and experts. Secondary sources (36%) include review articles or data in previously published databases, for which we could not access the primary source directly. For each GLOF, we recorded the name of the source lake and its parent glacier; the year and, if available, the month and day of occurrence; the elevation of the lake surface; and any previously gauged or estimated values of outburst peak discharge *Q*_p_ and flood volume *V*_0_. Using Bayesian hierarchical quantile regression models, we estimated global and regional trends in reported median and extreme discharges, defined as GLOFs exceeding the 50th and 90th percentiles of the conditional distributions of reported *Q*_p_ and *V*_0_. We also used regression models to assess changes in the regional timing and the median source elevation of ice-dam failures (Methods). We assessed the sensitivity of the posterior trends in GLOF magnitudes learned from data since 1900 against the period since 1990, as regional GLOF reporting improved in the late twentieth century^24^ (Extended Data Fig. 1).

## Less extreme GLOFs since 1900

**Fig. 2:**
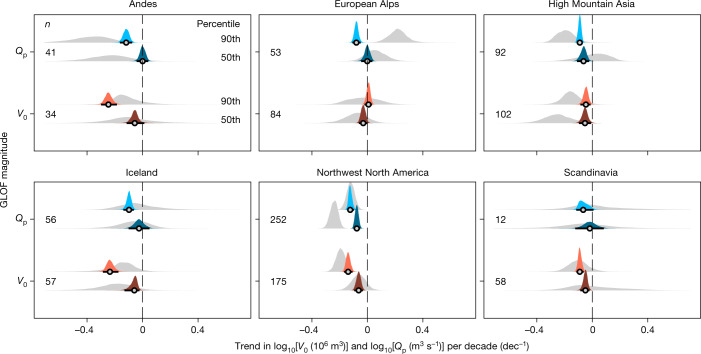
Trends of ice-dammed GLOF discharges between 1900 and 2021. Posterior trends from Bayesian quantile regression of reported *V*_0_ (orange) and *Q*_p_ (blue) with time. Dark and light colours are trends for the median and 90th percentile (that is, 10% largest), respectively, for 1900–2021; grey densities are trends for 1990–2021. Black lines are 95% HDIs and circles are medians of the posterior distributions. Trends refer to log_10_-transformed values of *V*_0_ and *Q*_p_, such that the posterior densities show the decadal trend in orders of magnitudes. Numbers (*n*) on the left are sample sizes for 1900–2021.

**Extended Data Fig. 2:**
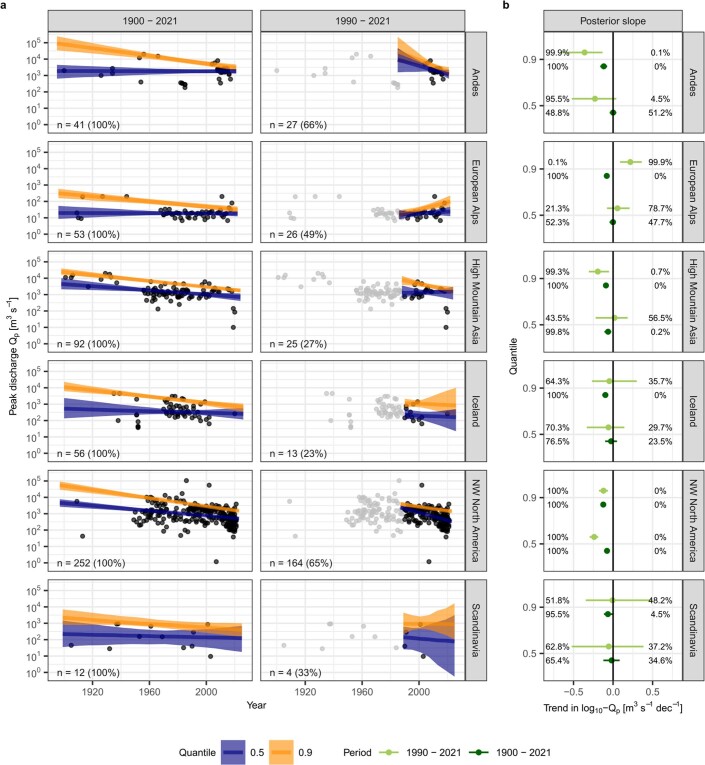
Regional trends of GLOF peak discharge *Q*_p_ with time *t* obtained by hierarchical quantile regression. **a**, Temporal trends of *Q*_p_ for the periods 1900–2021 and 1990–2021. Colours distinguish between the median (blue) and 90th (orange) percentile of reported *Q*_p_, including the posterior 95% HDI (shades). Numbers (n) and percentages in lower left of panels are, respectively, the sample sizes and shares of GLOFs used in the quantile regression model for each region. **b**, Posterior trends in *Q*_p_ for the 50th and 90th percentiles. Posterior 95% HDI (lines) and median (dot) regression trend of *Q*_p_ are grouped by the periods 1900–2021 (dark green) and 1990–2021 (light green). Distributions overlapping with zero (thick black line) indicate the lack of a credible trend in *Q*_p_. Percentages show posterior mass smaller (left) and larger (right) than zero.

**Extended Data Fig. 3:**
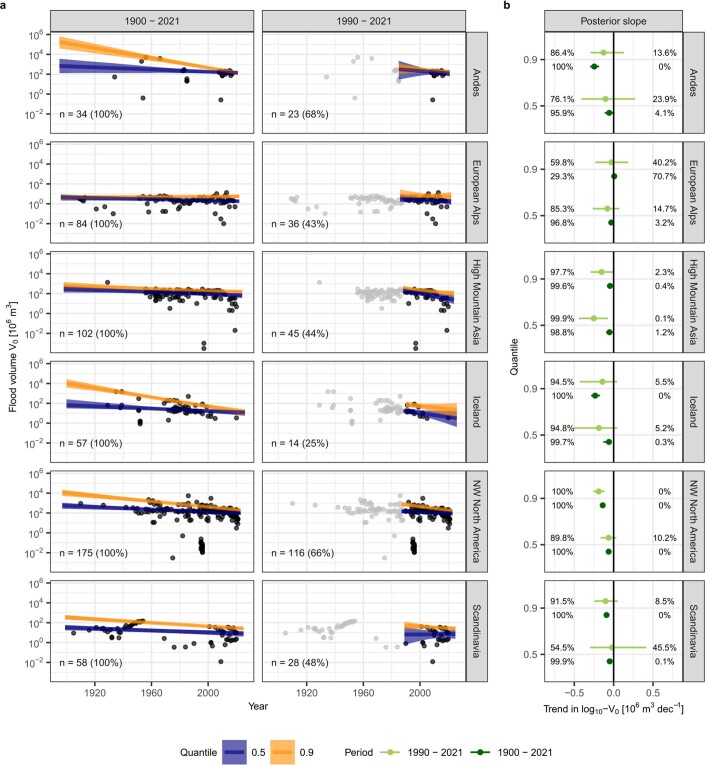
Regional trends of GLOF volume *V*_0_ with time *t* obtained by hierarchical quantile regression. **a**, Temporal trends of *V*_0_ for the periods 1900–2021 and 1990–2021. Colours distinguish between the median (blue) and 90th (orange) percentile of reported *V*_0_, including the posterior 95% HDI (shades). Numbers (*n*) and percentages in lower left of panels are, respectively, the sample sizes and shares of GLOFs used in the quantile regression model for each region. **b**, Posterior trends in *V*_0_ for the 50th and 90th percentiles. Posterior 95% HDI (lines) and median (dot) regression trend of *V*_0_, grouped by the period 1900–2021 (dark green) and 1990–2021 (light green). Distributions overlapping with zero (thick black line) indicate the lack of a credible trend in *V*_0_. Percentages show posterior mass smaller (left) and larger (right) than zero.

**Extended Data Fig. 4:**
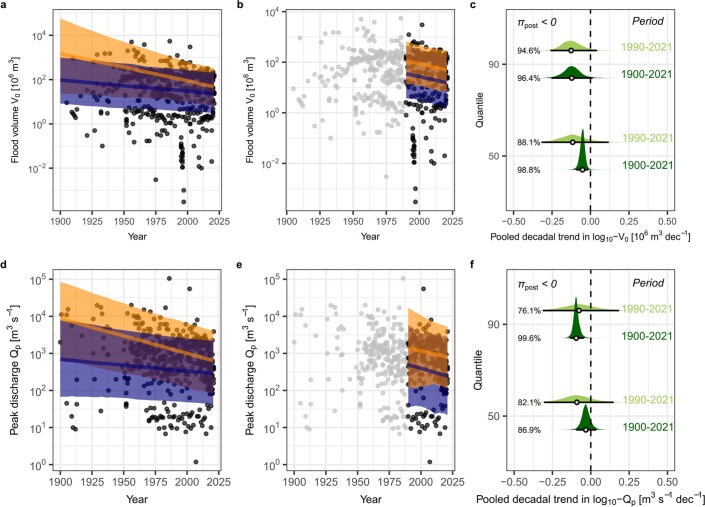
Global trends of GLOF volume *V*_0_ and peak discharge *Q*_p_ with time *t* obtained by hierarchical quantile regression. **a**, Pooled global trends in median and extreme *V*_0_ for the period 1900–2021. **b**, Pooled global trends in median and extreme *V*_0_ for the period 1990–2021. **c**, Posterior pooled trends in *V*_0_ for the 50th and 90th percentiles for both periods. **d**, Pooled global trends in median and extreme *Q*_p_ for the period 1900–2021. **e**, Pooled global trends in median and extreme *Q*_p_ for the period 1990–2021. **f**, Posterior pooled trends in *Q*_p_ for the 50th and 90th percentiles for both periods. In **a**, **b**, **d**, and **e**, colours distinguish between the median (blue) and 90th (orange) percentile of reported *V*_0_ and *Q*_p_, including the posterior 95% HDI (shades). In **c** and **f**, lines are the posterior 95% HDI and dots are the median of the global mean regression trends, grouped by the period 1900–2021 (dark green) and 1990–2021 (light green). Distributions overlapping with zero (dashed black line) indicate the lack of a credible trend in *V*_0_ and *Q*_p_. In **c** and **f**, *π*_*post*_ <0 denotes a posterior mass smaller than zero.

**Extended Data Fig. 5:**
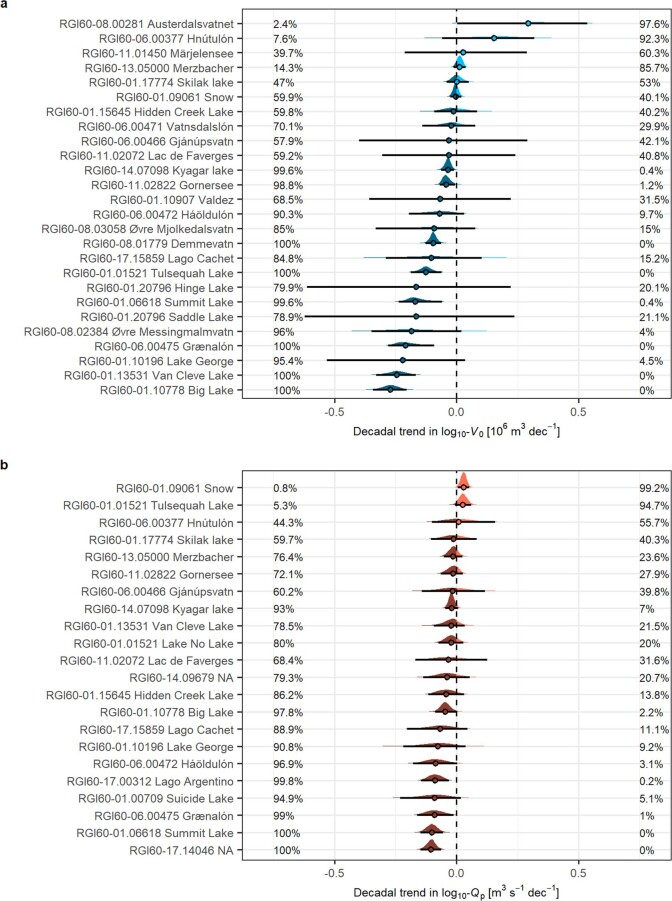
Posterior trends in local median (50th percentile) peak discharge *Q*_p_ and flood volume *V*_0_ obtained by hierarchical quantile regression. Posterior 95% HDI (lines) and median (dot) regression trend of **a**, *V*_0_ and **b**, *Q*_p_ of ice-dammed lakes. Glacier names are those of the parent glacier (RGI ID). Lakes labelled ‘NA’ have no name. Distributions overlapping with zero (dashed black line) indicate lack of credible trend in *V*_0_ and *Q*_p_. Percentages are posterior masses smaller (left) and larger (right) than zero. Trends refer to log_10_-transformed values of *V*_0_ and *Q*_p_. The posterior densities show decadal trends in orders of magnitudes.

Our inventory shows that the median reported discharge and volume released by ice-dammed lakes between 1900 and 2021 were $76{7}_{-756}^{+9,761}\,{{\rm{m}}}^{3}\,{{\rm{s}}}^{-1}$ and $5{1}_{-50}^{+1,100}\times 1{0}^{6}\,{{\rm{m}}}^{3}$, respectively (median and 95% highest density interval (HDI)). Runoff peaks more than ten times higher than this global median occurred in High Mountain Asia where glacier dams collapsed suddenly after phases of advance^3,7^. Dam failures following fjord closures in the Andes^25^ and Alaska^2^ produced floods with volumes exceeding 10^9^ m^3^. However, our quantile regression models suggest that extreme GLOFs have weakened over the past 120 yr (Fig. 2 and Extended Data Figs. 2–4). The 90th percentile decreased by at least an order of magnitude in five regions for *Q*_p_, and in four regions for *V*_0_. The Andes and northwest North America had a decline of almost two orders of magnitude in the extremes of *Q*_p_, whereas the highest *V*_0_ dropped even more in the Andes and Iceland. Only *Q*_p_ in Scandinavia and *V*_0_ in the European Alps had unchanged trends in the extremes. The regional medians remained largely stable since 1900: *Q*_p_ decreased credibly only in High Mountain Asia and northwest North America, without clear trends elsewhere. Four regions had a credible decrease in median *V*_0_, yet in most cases trends in median *V*_0_ were smaller than those of the extremes (Fig. 2 and Extended Data Fig. 3). A trend towards smaller floods is evident also for individual lakes with repeat (at least five) outbursts: hierarchical regression models show credible decreases in median *Q*_p_ in 23% (5 of 22), and in median *V*_0_ in 31% (8 of 26) of all cases. Only one lake had a credible increase in *Q*_p_ or *V*_0_ over the GLOF cycle (Extended Data Fig. 5).

All regions had more abundant reported GLOFs since 1990 compared with earlier decades (Extended Data Fig. 1). These changes in data density probably reflect a more consistent documentation of GLOFs^24^, such that an unknown number of lake outbursts may have gone unnoticed early in the study period. Reducing the observation period to the past three decades (1990–2021), a period with greater research activity, yields broader posterior trend estimates as fewer data points enter the model (Fig. 2). However, in almost all regions, the trends obtained from shorter periods are consistent with the long-term trends, even though based on less than two-thirds of the total data (Extended Data Figs. 2 and 3). Trends of extreme *Q*_p_ remain credibly negative in the Andes, High Mountain Asia and northwest North America, whereas median trends are unchanged in five of the six regions. Recent outbursts of Lac de Faverges (Switzerland) may explain the only increase in the 10% highest records of *Q*_p_ in the European Alps since 1990 (Fig. 2), although reported values of *Q*_p_ in this region are very low (<100 m^3^ s^−1^) by global standards^26,27^. Regional trends in extreme *V*_0_ since 1990 mirror those of extreme *Q*_p_, and are negative in five of the six regions. Except for a negative trend in High Mountain Asia, all regional trends in median *V*_0_ remain unchanged. By explicitly drawing on stratified subsets of the data, we conclude that regional trends in GLOF size over the recent shorter time interval with probably better documentation hardly deviate from the long-term trends. The recent decrease in extremes is striking because these arguably represent the best documented events with large values of *Q*_p_ and *V*_0_, making a potential increase in extreme GLOFs with time very unlikely.

## Earlier annual timing of GLOFs

**Fig. 3:**
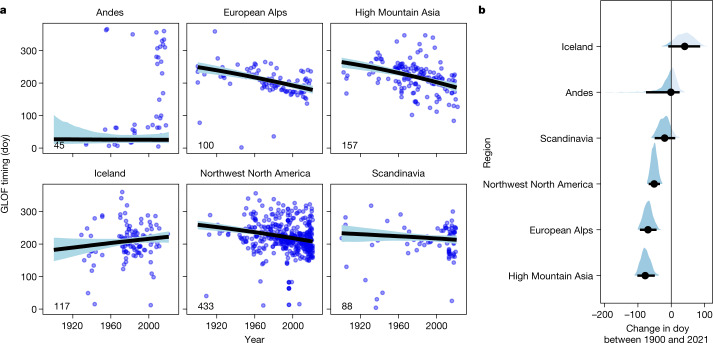
Changes in the annual timing of ice-dam failures between 1900 and 2021. **a**, Regional trends in the annual date of GLOFs. Thick black lines and light blue shades show, respectively, the median and 95% HDI of changes in the doy when glacier lakes burst out. Numbers in lower left corners are sample sizes. **b**, Posterior differences in GLOF timing (doy) between 1900 and 2021. Black bubbles are medians and horizontal lines are 95% posterior HDIs. Probability masses of negative trends are dark.

**Extended Data Fig. 6:**
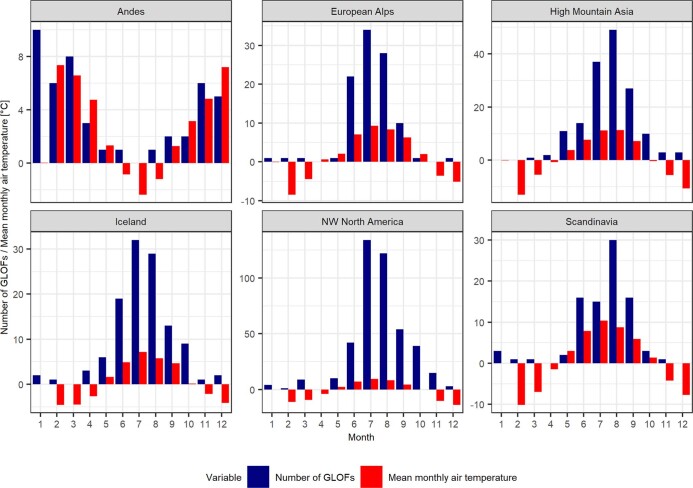
Regional seasonality of the monthly number of GLOFs and mean monthly air temperatures. Mean monthly air temperatures at the locations of all lakes that caused a GLOF during the period 1901–2021 (data from CRU TS V 4.05, ref. ^80^) were used to calculate the regional monthly average during that period. Months are sorted from 1 (January) to 12 (December).

**Extended Data Fig. 7:**
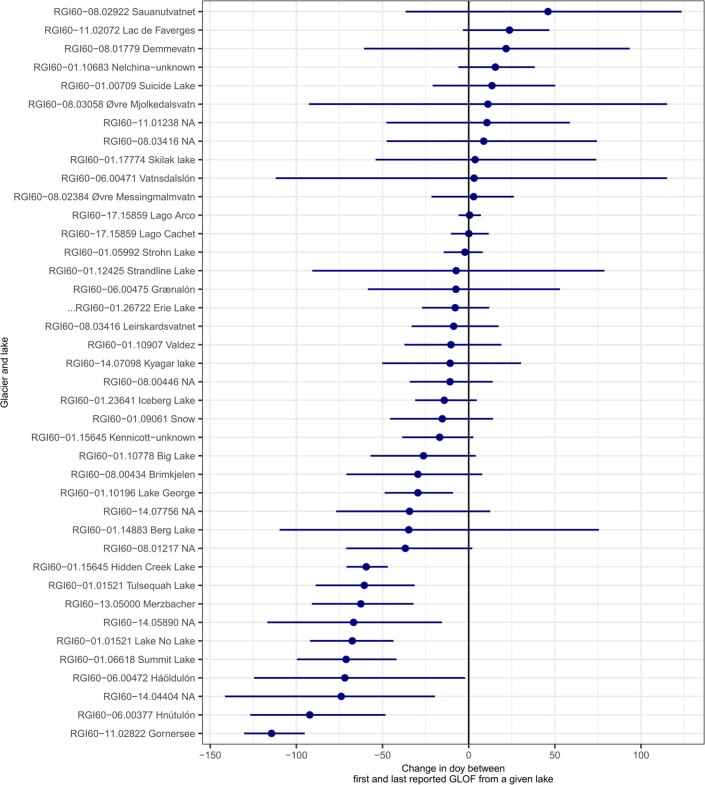
Posterior differences in GLOF timing (doy) for 40 ice-dammed lakes obtained by Bayesian regression. Lines and dots show, respectively, the posterior 95% HDI and median of the change in GLOF timing, expressed in days, between the first and the last reported GLOF from a given lake. Distributions overlapping with zero (thick vertical line) suggest no credible change in the day of year (doy). Lakes labelled ‘NA’ have no name.

Most GLOFs happen in summer, and winter remains largely free of GLOFs (Extended Data Fig. 6). In the Northern Hemisphere, GLOF activity peaks in July and August, and in the Southern Hemisphere in January when monthly average temperatures are highest, thus supplying ice-dammed lakes with large amounts of meltwater. Yet, the average date of outbursts within a given year has shifted earlier globally by about 40 days compared with 1900. The strongest regional shifts in timing are in High Mountain Asia ($7{7}_{-29}^{+25}$ days earlier), the European Alps ($7{0}_{-26}^{+24}$ days earlier) and northwest North America ($5{2}_{-19}^{+25}$ days earlier) (Fig. 3). In contrast, GLOFs in Iceland occur about six weeks later in the year on average, with the physical causes yet to be determined. At the site scale, we find that 68% of all (*n* = 40) ice-dammed lakes for which there is a documented day of the year (doy) burst out earlier in the year on average; in 30% of these cases, trends are credibly negative (Extended Data Fig. 7). The strongest change occurred at Lake Gornersee (Switzerland), which bursts out almost four months ($11{4}_{-19}^{+16}$ days) earlier today than at the beginning of the twentieth century^22^. Successively smaller and earlier floods could signal a local weakening, if not the end, of the GLOF cycle^21^. Indeed, already half (46) of the 101 glaciers with two or more failures stopped releasing GLOFs before 2000. Occasionally, human intervention interrupts the natural GLOF cycle, especially in cases where outbursts have repeatedly damaged infrastructure. In the European Alps and in Scandinavia, engineered structures such as drainage tunnels through bedrock or glacier ice stopped the recurrent outbursts of at least six ice-dammed lakes^28,29^.

## Increasing elevation of ice-dam failures

**Fig. 4:**
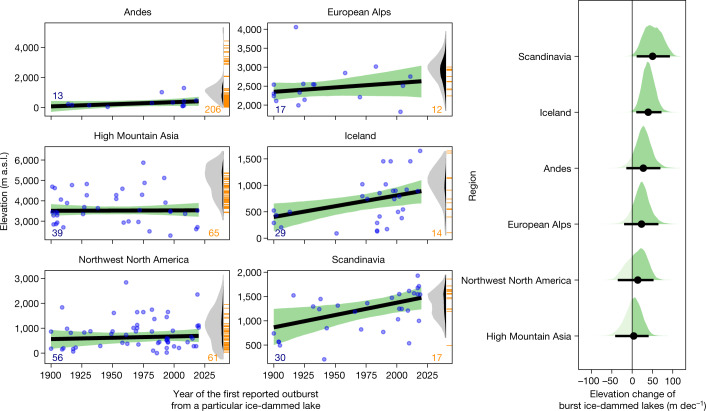
Elevation changes of GLOF sources. **a**, Changes in elevation of ice-dam failures over time. Blue bubbles mark years of the first reported outburst and the elevations above sea level (a.s.l.) of ice-dammed lakes. Thick black lines and green shades are the median and 95% HDI of the posterior trends, respectively. Blue numbers in lower left corners are the number of lakes that burst out between 1900 and 2021. Orange ticks and numbers on the right are elevations and the number of present-day ice-dammed lakes >0.01 km² in each region, respectively^48,55–58^. The grey densities estimate present-day glacier volumes across elevation from ref. ^47^, truncated at 95% of the data. The black areas within the grey densities show the loss of glacier volume over the period 2000–2019 from ref. ^8^, relative to the entire glacier volume. **b**, Posterior decadal trends in annual elevation change of GLOF sources. Black bubbles are medians and horizontal lines are 95% posterior HDIs. Probability masses of positive trends are dark.

**Extended Data Fig. 8:**
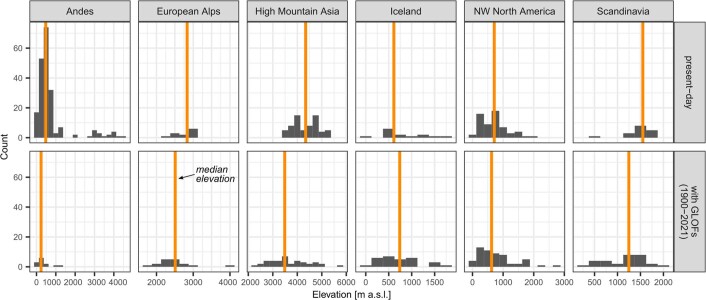
Regional elevation distribution of ice-dammed lakes. Histograms in top row show all lakes (>0.01 km²) that currently exist in the six regions. Those in the bottom row are all lakes that burst out between 1900 and 2021. Vertical orange lines are the median elevation of ice-dammed lakes in each region.

Our database further shows that outbursts from ice-dammed lakes originated at progressively higher elevations over the past 12 decades (Fig. 4). The largest elevation shifts are in Scandinavia, Iceland and the Andes, where new sources of GLOFs moved 25–50 m higher each decade on average. Outlet glaciers in these regions retreated rapidly in past decades, eliminating dams at their toes^25,30,31^. GLOFs in the European Alps had no credible change in elevation during our study period, although many large dams formed at lower elevations before 1900, when glaciers descended down to major valleys^2^. These dams disappeared with warming and glacier retreat after the mid-nineteenth century^2^. In contrast, High Mountain Asia has had more than 100 valley-blocking glacier advances to lower elevations in the past three decades^3,32^, explaining the unchanged elevation trend of ice-dam failures in that region. In most regions, outbursts emerged from lakes that were tens to many hundreds of metres lower in elevation than today (Fig. 4 and Extended Data Fig. 8). In three regions, the number of ice-dammed lakes decreased over time: Scandinavia, the European Alps and Iceland have, in total, only 25 ice-dammed lakes >0.01 km^2^ today, whereas three times as many lakes burst out in the past 120 yr (Fig. 4).

## Impact of glacier decay on GLOF size

**Fig. 5:**
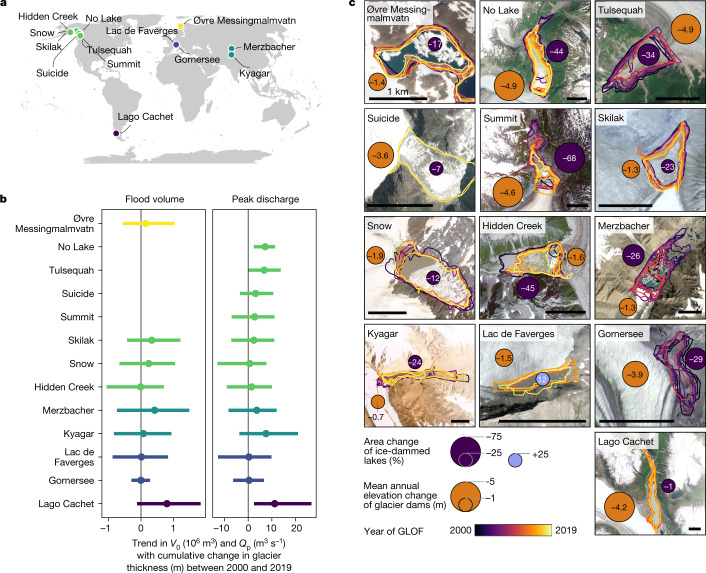
Trends in flood volume, peak discharge and lake area with cumulative changes in glacier thickness between 2000 and 2019. **a**, Map of 15 ice-dammed lakes that produced more than five GLOFs each in that period. We generated the map in the free software RStudio V2022.07.2 (https://posit.co/products/open-source/rstudio/) using the ggmap package, including free data on administrative boundaries from the rnaturalearth package. **b**, Posterior regression slopes of *V*_0_ (left) and *Q*_p_ (right) versus cumulative change in thickness of the ice dam. Bubbles are medians and horizontal lines are 95% posterior HDIs of the posterior trends. Colours in **a** and **b** distinguish the study regions. Missing trends for individual lakes arise from lack of data. **c**, Local changes in lake area and glacier elevation between 2000 and 2019. We mapped lake areas (polygons) from satellite imagery immediately before the outburst using QGIS V3.22.6. Colours show the year of the GLOFs. Blue bubbles show the average percent change in lake area between the first and last reported GLOF for each lake. Orange bubbles show the mean annual elevation change of the glacier dam. Black horizontal scale bar is 1 km in all panels in **c**. Data on lake and glacier elevation change are available at ref. ^27^. All background imagery are from 2019 Planet Labs Inc., obtained under a basic license in the Education and Research programme. We generated all maps in **c** using QGIS V3.22.6.

**Extended Data Fig. 9:**
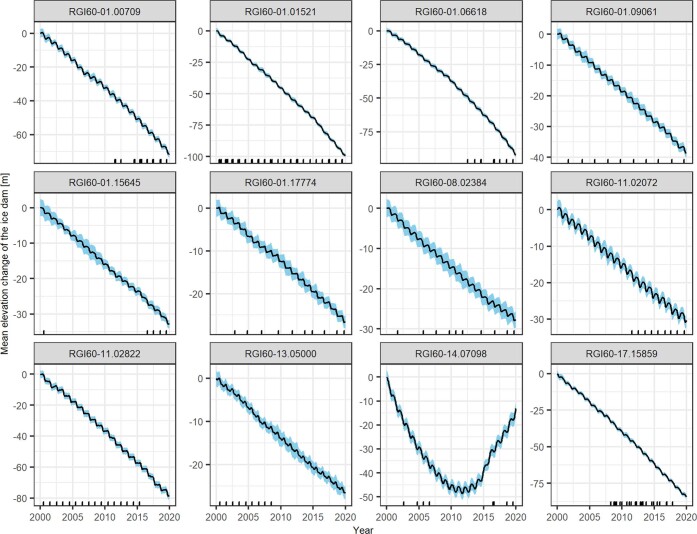
Mean elevation change of glacier dams with reported GLOF magnitudes between 2000 and 2019. Black line and blue shading are, respectively, the mean and standard error of glacier elevation change obtained by Gaussian Process regression^8^. Black ticks are dated GLOFs. Note that Tulsequah Glacier (RGI60-01.01521) dams two lakes. The reversed trend for Kyagar Glacier (RGI60-14.07098) is due to a glacier surge.

How does overall glacier decay help explain changes in ice-dam failures? In theory, the drainage of ice-dammed lakes occurs when the water pressure in the lake is close to or exceeds the pressure of the adjacent ice dam^33–35^. A GLOF may initiate once a critical ratio between lake depth and glacier thickness is reached^20^. As glaciers thin due to atmospheric warming^8^, the necessary water depth to trigger a GLOF should lower, resulting in smaller lakes that produce floods of decreasing magnitudes^20^. We searched for a possible correlation between glacier thinning and GLOF magnitudes by focusing on 12 glaciers (one glacier impounds two lakes), for which we could obtain the cumulative elevation change of the ice dam, maximum lake areas before outbursts and reported GLOF magnitudes (*V*_0_ or *Q*_p_) between 2000 and 2019 (Methods). Over this period, we observed a thinning of the glacier dams by ${36}_{-62}^{+19}\,{\rm{m}}$ (median and 95% HDI; Fig. 5c and Extended Data Fig. 9), and that 12 of 13 lakes shrank, with 8 losing more than 20% of their surface areas (Fig. 5c). Yet, rapid glacier thinning appears to correlate only moderately with reported GLOF magnitudes: average trends between glacier thinning and decreases in flood volumes or peak discharges are positive for only half of the glacier dams (Fig. 5a,b). Credibly positive trends occur only at Tulsequah Lake and Lake No Lake (British Columbia), and at Lago Cachet (Patagonia). Most other lakes have no clear trend. Interactions between ice and water might be an important mechanism for removing parts of glacier dams, causing most ice-dammed lakes to shift laterally towards the glacier dam during the GLOF cycle. For example, Lake No Lake and Summit Lake (northwest North America) eroded several hundreds of metres from their ice dams in the past 20 yr (Fig. 5c).

The unclear relationship between changes in glacier thickness and GLOF magnitudes suggests a nonlinear response, or instead that glacier thinning is a poor proxy for GLOF magnitudes. Variances among the estimated local trends point to local drainage mechanisms. Small variances in *Q*_p_ and *V*_0_ (ref. ^27^) may indicate recurrent opening and closing of the same subglacial tunnel system that allows the floods to attain high peak flows despite decreases in glacier thickness^22^. Floating ice can further exert high pressures on the underlying water in lakes, maintaining high *Q*_p_ and *V*_0_ independent of glacier thinning that reduces the basin’s storage capacity^36^. In contrast, high variances or abrupt changes in the trends may indicate shifts between drainage regimes, for example due to failure of ice buttresses at the point of inflow into the glacier^19^. Ice motion during an outburst can also close subglacial tunnels before the lake has emptied completely^34^. Some lakes such as Lago Cachet (Fig. 5c) have maintained their surface area but gained depth as sediment was removed from the lake bottom between successive outbursts^12^. Outburst floods can also drain other water bodies within and beneath the glacier. The volume from these sources can rival or even exceed those of the subaerial lake, although distinguishing the contributions from the individual sources is subject to high uncertainties^37,38^. Local weather can also add to the observed variability. Water supply to lakes can suddenly increase following heavy precipitation or during heatwaves when there is high glacier and snow melt, resulting in unusual high peak flows^16,39^. Accurately determining such local water fluxes could make GLOFs more predictable^22,37,40^, although it would require a much denser meteorological network to quantify weather conditions in ice-dammed basins^41^.

## Implications for GLOF hazard

In our study regions, >70% of all reported GLOFs since 1900 originated from ice-dammed lakes^24^. However, their present-day number and sizes are small compared with meltwater lakes dammed by moraines or bedrock. In northwest North America, ice-dammed lakes account only for 6% of the total lake abundance^18^, and in High Mountain Asia, as few as ~0.06% of total lake area is impounded by glaciers^42^. However, given their high outburst activity, we infer that ice-dammed lakes are among the most hazardous of all meltwater lakes. Still, catastrophic damage and fatalities remain a rare consequence of ice-dammed failures: only 10% of all floods in our database have documented socio-economic impacts, mostly involving destroyed bridges, pipelines, roads and campgrounds buried in debris, or loss of farmland and cattle affecting local food supply. Agricultural loss prevails in reports from Scandinavia, Iceland, the Andes and the European Alps. Since 1900, three ice dam failures have claimed human lives, although loss of life was reported more frequently in previous centuries. Well documented cases include the sudden failures of an ice-dammed lake in the Dranse Valley, Swiss Alps, in 1590 and 1818 that killed at least 40 and 140 people, respectively^43^. Such one-time ice-dam failures and those starting a new, unexpected GLOF cycle with flood defence measures not yet in place have caused much damage^16,44^. Dykes, floodgates, dams, or the temporary or permanent resettlement of mountain communities have reduced the impact of recurrent ice-dam failures, even for exceptionally large floods^1^.

The global reduction in glacier volumes^8^ will probably control ice-dam failure in the future. We find that 90% of all outburst floods in our database originated below the elevation band where most glacier mass is stored today (Fig. 4a). Elevations of historical ice-dam failures are largely within glacier ablation areas, where glaciers melt fastest and temporarily trap excess meltwater at their margins. This zone has been rapidly shifting upwards under ongoing atmospheric warming. In the European Alps and the Himalayas, for example, glaciers have been melting throughout their entire elevation range in the twenty-first century^45,46^, and ice-dammed lakes are now forming even in the highest parts of ice-covered catchments (Fig. 4a and Extended Data Fig. 8). Progressive retreat at the glacier terminus may thus push the accommodation space for new lakes towards the natural limit set by the remaining glacier mass close to the drainage divide. According to projections, regions with small amounts and volumes of glaciers, such as the European Alps, Scandinavia and British Columbia, could become largely ice free by 2100^9^. We anticipate that the window for future ice-dam formation and failure might close within a few decades in these mountain ranges. Glacier remnants in steeper and higher terrain may be capable of only damming, if at all^5^, smaller lakes, albeit with higher potential energy. In the course of one to several centuries, ice-dam failures may diminish as a cryospheric hazard, although mass flows from other lakes, possibly tied to permafrost thaw, may take over.

This trajectory of changing GLOF hazard is likely to differ in regions with large glacier volumes. Alaska and Patagonia, for example, host ~70% of the total glacier volume in our study regions, about half of which will persist beyond the end of the twenty-first century^9,47^. Extensive glacier streams currently terminate at or near sea level, and hundreds of glacier-dammed lakes have been forming in ice-free tributary valleys in recent decades^18,48^ (Fig. 4a). Yet, statistical models have failed to identify glaciological or meteorological predictors that could explain the growth pattern of ice-dammed lakes in northwest North America^49^. Trends in the magnitude and timing of GLOFs, let alone any drivers, are largely unknown for most of these lakes^23^. Newly exposed proglacial areas could be sources of new cycles of GLOFs, posing hazards for potential development in tourism, mining and hydropower generation in these areas. In this regard, time series of satellite images^50^ and stream gauges^12,14,22^ are invaluable archives to systematically capture geomorphic changes caused by GLOFs. Integrating newly detected and reported cases into a single database is thus key to quantifying and predicting climate-driven changes in GLOF magnitude, frequency and hazard^24^.

In summary, our analysis reveals a strong decay in the highest GLOF magnitudes and discharges, whereas GLOFs of median size have changed little in the past 12 decades. Given their steep hydrographs, floods caused by ice-dam failures will remain discharge extremes, even during glacier retreat. How and where the remaining glaciers will form dams in the future will determine the extent to which GLOFs will feed the sediment cascade^12^, disturb ecological and geochemical cycles in mountain rivers^51,52^, and locally inundate floodplains and terraces^7^. To anticipate these consequences, future work will be required to refine the few available physical models that link glacier retreat and lake formation with the size and timing of outbursts^10,34–36^. Recently released high-resolution data on elevation, glacier change, ice-flow velocities and climate will be key to understanding better the nexus between meltwater accumulation and hazard. Predicting and monitoring the locations and dynamics of future lakes will become increasingly important, especially in steep mountain regions that are difficult to access, but have growing populations and infrastructure downstream. International collaborations such as the United Nations Development Programme project ‘GLOF-I/-II’ in Pakistan aim to mitigate GLOF hazards, but more effort will be required to manage the episodic GLOF discharges as the variability in precipitation, temperature and river discharge rises^53,54^. Our dataset forms a solid base to help predict changes in GLOF hazard from the catchment to the regional scale to ensure more sustainable development for one of the most sensitive regions on Earth.

## Methods

### Developing a database of ice-dam failures

We define an ice-dammed lake as any water body at the margin of and impounded by glacier ice. We exclude all other glacier-fed lakes, such as lakes dammed by moraines or landslides, internal water pockets, supraglacial ponds, lakes below glaciers and lakes formed by geothermal activity. Ice dams often trap runoff from catchments and cause loss and damage when the impounded water is suddenly released and reaches human settlements^59^. We focus on six major glaciated mountain regions that support 99% of the contemporary ice volume outside polar regions^47^. Our evidence for the 1,569 ice dam failures reported between 1900 and 2021 comes from a total of 446 different sources of information. We distinguish between primary sources, where we have direct observations or access to raw data, and secondary sources that describe, interpret or summarize previous findings on historical GLOFs. Primary sources are available for 64% of all reported cases and include original scientific publications (36%), annals and reports published by local and regional authorities (18%), written communications in emails with eyewitnesses and experts (9%), and newspapers and internet resources such as video footage and posts on social media platforms (altogether 1%). Secondary sources (36% of all cases) include second-hand information derived from reviews and summaries of previous publications (19%) and entries in databases with no direct access to the primary source (17%). Half of all sources report only a single event, whereas a few studies include information on dozens of GLOFs. For example, ref. ^60^ identified more than 100 outbursts from lakes impounded by Kennicott Glacier during the twentieth century by studying stream gauge data and historical aerial photographs. Still, decades may elapse between the date of a GLOF and the reporting, especially where researchers reprocessed and reanalysed historical data (Extended Data Fig. 1). In half of all cases, however, GLOFs were reported within a decade after they occurred. A few studies contributed significantly to improving the regional record of GLOFs in the first decades of the twentieth century, when the overall reporting rate was low^3,7,11,60–62^. In a few cases, reporting may have changed due to resettlement in this period. For example, repeated flooding of an ice-dammed lake below the Folgefonni Ice Cap (Norway) forced farmers to abandon their homes in the mid-1960s^16^. It is only since the 1970s that research activity on GLOFs has become more consistent^24^: 63% GLOFs in our databases were first documented in that period (Extended Data Fig. 1).

In collecting historic GLOFs, we only considered cases with either a documented date (at least the year of occurrence) or a time interval of failure, centroidal coordinates of the lake and at least one reference. For each GLOF, we documented the mountain range, the country from which the flood originated, the name of the parent glacier (local name and the ID in the Randolph Glacier Inventory RGI V6.0^63^) and the name of the burst glacier lake. We extracted the surface elevation of each lake centroid from the Advanced Land Observing Satellite (ALOS) V3.2 digital elevation model (DEM)^64^. We collected the reported flood volume (*n* = 510, 33% of all cases), peak discharge (*n* = 506, 33%) and timing (*n* = 940, 61%). Finally, we documented any impacts, damage, and losses for events in our database.

### Lake mapping

We could verify the origin, timing and area of 53% of all reported lake outbursts that have occurred since the Landsat 5 mission began to continuously obtain satellite images in the late-1980s^65^. Later generations of satellites, such as RapidEye and the Planet Cubesat constellation (launched in 2009 and 2015, respectively), have higher spatial resolution and thus capture GLOF features in greater detail. Ice-dammed lakes decrease in size, or even empty entirely, during GLOFs, exposing either partly or fully the lake floor, which is often covered with stranded icebergs (Fig. 5c). Ice, clouds, and shadows compromise the automatic detection of water and lakes^66^. Hence, we manually digitized the extents of the lakes from satellite images in QGIS V3.16 software. To this end, we scanned the archive of satellite images to retrieve the last available image before and the first available image after the lake drained. Mapping lakes from optical images was limited in Iceland because of frequent cloud cover. For each lake with repeat outbursts, we used a simple linear regression model of the mapped lake area before the outbursts versus time to obtain the change in lake area (Fig. 5). We then estimated the mean percent change in lake area between the first and last reported outburst in the time series. For manually mapped lake areas, uncertainties are generally assumed to be between 4% and 10% of the mapped lake area^48,55^ or between 0.5 and 1 pixel of the sensor resolution^18,42,57^, that is, 3–5 m for Planet and RapidEye, and 30 m for Landsat images.

In Fig. 4, we compare elevations of lakes that have had historic outbursts with elevations of ice-dammed lakes that currently (2018–2020) exist according to regional inventories^18,48,55–58^. The lakes in these databases were either manually digitized or refined after automatic mapping. We chose a minimum mapping unit of 0.01 km^2^ to ensure comparability between inventories. No contemporary lake inventory is available for Iceland. Thus, we manually mapped ice-dammed Icelandic lakes from high-resolution Google Earth imagery obtained between May and September 2021. We added the elevations obtained from the ALOS V3.2 DEM^64^ to both the previous lake inventories and our manually mapped lakes.

### Bayesian hierarchical modelling

To assess temporal changes in GLOF magnitude, elevation and timing, we fitted Bayesian hierarchical models that distinguish between regional (that is, our six study regions) or local (that is, lakes with repeat outbursts) groups. Bayesian models form a compromise between the likelihood of observing the data under the assumption of specific model parameters and prior knowledge about these parameters. We encode our uncertainty in this prior knowledge before obtaining a posterior distribution for all parameters conditioned on the data. The benefit of hierarchical models is that they account for group-level structure in the data within a single model. We can thus estimate regional or local effects with respect to a population-level model learned from all data regardless of their location. Hierarchical models offer a compromise between separate models that overemphasize regional and local effects and that are independently learned for each group, and pooled models that are learned from all data without distinction^67^. Hierarchical models improve parameter estimates for individual groups, especially if sample sizes are low or vary across the groups. This trait is particularly desirable for our study as the number of reported GLOFs differs widely among both regions and lakes. The model learns the population-level parameters from the data, and these parameters are shared among groups. In this way, the groups inform each other, achieving an effective sample size that greatly exceeds that of single groups. The joint posterior density in a hierarchical model is proportional to the product of the likelihood and the joint prior density $$\pi ({\theta }_{1},\ldots ,{\theta }_{J},\tau {|D})\propto $$$$\pi ({D|}{\theta }_{1},\ldots ,{\theta }_{J},\tau )\pi ({\theta }_{1},\ldots ,{\theta }_{J},\tau )=\pi (\tau ){\prod }_{\{j=1\}}^{J}\pi ({D}_{j}|{\theta }_{j})\pi ({\theta }_{j}|\tau )$$, where *D* are the observed data and *D*_*j*_ is the subset of *D* that contains all data in group *j*. The group-level parameters $${\theta }_{j}=({\theta }_{1j},\ldots ,{\theta }_{{Kj}})$$ define the model on the group-level with *K* individual parameters, which vary for $$j=1,\ldots ,J$$ groups. These parameters are drawn from distributions $$\pi \left({\theta }_{j}|\tau \right)$$ and specified by population-level (hyper-) parameters $$\tau =\left({\tau }_{1},\ldots ,{\tau }_{L}\right)$$, which consist of *L* individual parameters and are also learned from the data. The hierarchical model estimates the posterior distribution for both population-level and group-level parameters conditional on the reported GLOFs.

### Assessing regional and local trends of *V*_0_, *Q*_p_ and *Z*

Continental and global studies of river floods point to climate-driven changes in both median and extreme discharges in past decades^68–71^. Similarly, repeated inventories reveal recent shifts in the regional size distribution of ice-dammed lakes, but little about trends in lake outbursts^18,48,49^. By using quantile regression, we examined whether and by how much the quantiles in the conditional distributions of reported peak discharge, *Q*_p_, and flood volume, *V*_0_, have changed in our six study regions. Extreme (>90th percentile) flood discharges are important for planning flood protection measures and informing flood risk zoning^69^. Ordinary least squares regression estimates the conditional mean, but quantile regression makes no assumptions about the distribution of the response variable and is robust against outliers. In our model specification, we used an asymmetric Laplace likelihood (AL) with fixed quantiles. The AL has density

1 $${f}_{{\rm{AL}}}(\,y{\rm{| }}\mu ,\kappa ,p)=\frac{p(1-p)}{\kappa }\exp \left(-{\rho }_{p}\left(\frac{y-\mu }{\kappa }\right)\right),$$

where *y* is the response variable, *μ* is a location parameter, *κ* is a positive scale parameter, 0 < *p* < 1 is a percentile, and ${\rho }_{p}(x)=x(p-I(x < 0))$ with indicator function *I*(∙). The exponent takes the values $-\frac{\,|{y}-\mu |}{\kappa }p$ for *y* ≥ *μ* and $-\frac{|y-\mu \,|}{\kappa }(1-p)$ for *y* < *μ*. We modelled the conditional distributions of *Q*_p_ and *V*_0_ with decimal year *t* for two fixed values of *p* as

2 $${y}_{ji}\sim {\rm{A}}{\rm{L}}({\mu }_{ji},\kappa ,p),{\rm{f}}{\rm{o}}{\rm{r}}\,j=1,\ldots ,\,J\,{\rm{a}}{\rm{n}}{\rm{d}}\,i=1,\ldots ,{n}_{j}$$

3 $${{\rm{\mu }}}_{ji}={\alpha }_{j}+{\beta }_{j}{t}_{ji},{\rm{for}}\,j=1,\ldots ,\,J\,{\rm{and}}\,i=1,\ldots ,{n}_{j}$$

4 $$[\begin{array}{c}{\alpha }_{j}\\ {\beta }_{j}\end{array}]\sim {\rm{M}}{\rm{V}}{\rm{N}}{\rm{o}}{\rm{r}}{\rm{m}}{\rm{a}}{\rm{l}}\,[(\begin{array}{c}\alpha \\ \beta \end{array}),S],\,{\rm{f}}{\rm{o}}{\rm{r}}\,j=1,\ldots ,J$$

5 $$S=\left(\begin{array}{cc}{\sigma }_{\alpha } & 0\\ 0 & {\sigma }_{\beta }\end{array}\right)R\left(\begin{array}{cc}{\sigma }_{\alpha } & 0\\ 0 & {\sigma }_{\beta }\end{array}\right)$$

6 $$R=\left(\begin{array}{cc}1 & \varsigma \\ \varsigma  & 1\end{array}\right)$$

where *y*_*ji*_ are reported values of *Q*_p_ or *V*_0_ with *y*_*ji*_ referring to the *i*th observation in group *j*, *n*_*j*_ is the number of observations in group *j*, and *J* is the number of groups. The parameters *α*_*j*_ and *β*_*j*_ are the group-level intercepts and slopes, respectively, and *α* and *β* are the corresponding population-level parameters. The covariance matrix *S* is composed of group-level standard deviations *σ*_*α*_ and *σ*_*β*_, and *R*, the correlation matrix with correlation *ς*. In line with decadal analyses of median and extreme flood discharges^68–70^, we considered fixed values of *p* = 0.5 (median) and *p* = 0.9 (90th percentile), and estimated all other parameters from the data.

The Bayesian framework demands prior distributions for each parameter that enters at another model level. We chose the following priors

7 $$\kappa \sim N(0,2.5)$$

8 $$\alpha \sim N(0,2.5)$$

9 $$\beta \sim N(0,2.5)$$

10 $${\sigma }_{\alpha }\sim N(0,2.5)$$

11 $${\sigma }_{\beta }\sim N(0,2.5)$$

12 $$R\sim {\rm{L}}{\rm{K}}{\rm{J}}{\rm{C}}{\rm{h}}{\rm{o}}{\rm{l}}{\rm{e}}{\rm{s}}{\rm{k}}{\rm{y}}(1).$$

These priors refer to standardized, log_10_-transformed response variables *Q*_p_ and *V*_0_, and a standardized predictor (decimal year of observation) with zero mean and unit standard deviation. Our choice of a zero-mean Gaussian with standard deviation of 2.5 admits both negative and positive trends for *β*, expressing the contrasting trends reported for the size of ice-dam failures in recent decades^1,6,19,22,72^. The Lewandowski–Kurowicka–Joe (LKJ) Cholesky correlation distribution prior for *R* makes all correlation matrices equally likely.

For a fixed quantile, the joint posterior distribution can be thus written as

13 $$\begin{array}{c}\pi ({\alpha }_{1},...,{\alpha }_{J},{\beta }_{1},...,{\beta }_{J},\kappa ,\alpha ,\beta ,{\sigma }_{\alpha },{\sigma }_{\beta },R|y,t,p)\propto \pi (\kappa ,\alpha ,\beta ,{\sigma }_{\alpha },{\sigma }_{\beta },R)\\ \,({\prod }_{j=1}^{J}{\prod }_{i=1}^{{n}_{j}}{f}_{{\rm{A}}{\rm{L}}}(\,{y}_{ji}|{\alpha }_{j}+{\beta }_{j}{t}_{ji},p,\kappa )\pi ({\alpha }_{j},{\beta }_{j}|\alpha ,\beta ,{\sigma }_{\alpha },{\sigma }_{\beta },R)),\end{array}$$

where $\pi \left({\alpha }_{j},{\beta }_{j}| \alpha ,\beta ,{\sigma }_{\alpha },{\sigma }_{\beta },R\right)$ is the prior distribution for the group-level parameters as defined in equations (4)–(6) and $\pi \left(\kappa ,\alpha ,\beta ,{\sigma }_{\alpha },{\sigma }_{\beta },R\right)$ is the joint prior distribution for the independent population-level parameters as defined in equations (7)–(12). We numerically approximate the posterior distribution using a Hamiltonian sampling algorithm implemented in Stan^73^ that is called via the software package brms^74^ within the statistical programming language R^75^.

We used the same multi-level structure of the quantile regression model to determine how flood discharges have changed for individual lakes. To this end, we conditioned the model on *J* = 22 lakes (*Q*_p_) and *J* = 26 lakes (*V*_0_) that produced at least five outbursts each between 1900 and 2021. For these models, we focused only on the trends in median flood discharges, given that lakes with few reported GLOFs have few samples that exceed high thresholds.

Finally, to test whether more recent GLOFs originated from progressively higher elevations, we selected for each lake the year of the first reported GLOF, given that many ice-dammed lakes burst out repeatedly. As for our hierarchical models on GLOF magnitudes, we used quantile regression to determine the trend in the median elevation *Z* of glacier-dammed lakes against the decimal year of GLOF occurrence *t*, conditioned on the *J* = 6 regions.

We ran all regional and local quantile regression models on *Q*_p_, *V*_0_ and *Z* with three parallel chains that drew 6,000 samples with 2,000 warm-up runs each. We found no numerical divergences after warm-ups, which were supported by the Gelman–Rubin potential scale reduction factor $\hat{R}=1.0$ in all models, indicating that the Markov chains have converged. We report the posterior distributions of the pooled model parameters in Supplementary Tables 1–3, and the group-level regression slopes in Extended Data Figs. 2, 3 and 5. These regression slopes are conventionally deemed ‘credibly’ different from zero if a select interval of the posterior probability mass, in our case the 95% HDI, excludes zero. This choice of the HDI is arbitrary, and we also report how much of the probability mass of the posterior distribution is on either side of zero (Extended Data Figs. 2–5).

### Assessing trends in doy

Measuring temporal changes in GLOF timing must consider that the response variable doy can recycle through the observation period, given that calendar day 0 is equal to day 365. The von Mises (VM) distribution is a close approximation to a wrapped normal distribution for circular data and allows estimating the angular response *d* (doy) from the linear predictor year *t* (ref. ^64^). The VM distribution has density

14 $${f}_{{\rm{VM}}}(\,y| {\vartheta },\phi )=\frac{{\rm{\exp }}(\phi \,\cos (\,y-{\vartheta }))}{2\pi {I}_{0}(\phi )},$$

where *y* are the observed data, *ϑ* is a location parameter, *φ* is a positive precision parameter and *I*_0_ is the modified Bessel function of the first kind of order 0.

Following the parameterization in brms, we rescaled the response *d* to ${y}_{i}\in (-\pi ,\pi )$, standardized *t* to a zero mean and unit standard deviation, and estimated the trend in GLOF timing as

15 $${y}_{ji}\sim {\rm{V}}{\rm{M}}({\vartheta }_{ji},{\phi }_{j}),\,{\rm{f}}{\rm{o}}{\rm{r}}\,j=1,\,\ldots ,\,J\,{\rm{a}}{\rm{n}}{\rm{d}}\,i=1,\,\ldots ,\,{n}_{j}$$

16 $${{\vartheta }}_{ji}={\zeta }_{j}+{\eta }_{j}{t}_{ji},\,{\rm{for}}\,j=1,\,\ldots ,\,J\,{\rm{and}}\,i=1,\,\ldots ,\,{n}_{j}$$

17 $${\zeta }_{j}\sim N(0,2.5)$$

18 $${\eta }_{j}\sim N(0,2.5)$$

19 $${\phi }_{j}\sim {\rm{G}}{\rm{a}}{\rm{m}}{\rm{m}}{\rm{a}}(2,0.01),$$

where *y*_*ji*_ are reported values of doy, with *y*_*ji*_ referring to the *i*th observation in group *j*, *ϑ*_*j*__*i*_ is the corresponding location parameter, and *ζ*_*j*_ and *η*_*j*_ are the estimated intercepts and slopes, respectively. We used the default Gamma prior in brms to estimate the precision parameter *φ*_*j*_. This model was fit to each group separately without partial pooling of parameters across groups, because the distinct seasonal differences in GLOF timing in the two hemispheres (Extended Data Fig. 6) caused numerical divergences. Using a VM likelihood function, the joint posterior distribution can be written as

20 $$\begin{array}{c}\pi ({\zeta }_{j},{\eta }_{j},{\phi }_{j}|{y}_{j},{t}_{j})\propto ({\prod }_{i=1}^{{n}_{j}}{f}_{{\rm{V}}{\rm{M}}}({y}_{ji}|{\zeta }_{j}+{\eta }_{j}{t}_{ji},{\phi }_{j}))\,\pi ({\zeta }_{j},{\eta }_{j},{\phi }_{j}),\\ \,\,\,{\rm{f}}{\rm{o}}{\rm{r}}\,j=1,\,\ldots ,\,J\end{array}$$

where *y*_*j*_ and *t*_*j*_ are the observations and corresponding years of group *j* and $\pi ({\zeta }_{j},{\eta }_{j},{\phi }_{j})$ is the joint prior of the regression parameters. We maintained the set-up of the Hamiltonian sampler in brms, using three parallel chains each with 6,000 samples after 2,000 warm-up runs. We found that $\hat{R}=1.0$ in all runs, indicating convergent Markov chains. We report the posterior distributions of all model parameters in Supplementary Table 4.

### Estimating local trends between glacier elevation change and *V*_0_ and *Q*_p_

The length and thickness of the glacier dam are important diagnostics for predicting flood magnitudes and timing^76^. Changes in dam geometry could therefore alter the pattern of lake outbursts, in both frequency and magnitude. In QGIS, we manually trimmed the area of all glaciers in the RGI V6.0 that impound a lake to the area below the dam. We define a dam as the area between the glacier terminus and the elevation contour on the glacier directly upstream of the lake. We then extracted the cumulative elevation change of all glacier dams between 2000 and 2019, following the procedure by ref. ^8^. In summary, this method first generates DEMs from all available stereo-pairs of the Advanced Spaceborne Thermal Emission and Reflection Radiometer (ASTER) satellite instrument intersecting glaciers during the period of 2000 to 2019. We stacked the ASTER DEMs in temporal order and further added high-resolution data from the ArcticDEM^77^ to the time series. Data from ArcticDEM cover glacier dams in Alaska, Iceland and Scandinavia (Supplementary Table 6). Each pixel in the DEM time series is then interpolated using a Gaussian process regression model to estimate the elevation at any date within this period. We extracted the elevation time series for the areas of the glacier dams, resulting in an area-weighted average of 45 independent DEMs between 2000 and 2019 for each dam. Individual glacier dams are covered by at least 95% of measured pixels, drawing on at least 13 independent DEMs per pixel. We calculated the associated elevation differences between successive time steps, interpolated spatial data gaps hypsometrically^78^ and finally aggregated them to the cumulative mean elevation changes with respect to 1 January 2000. On average, our glacier dams thin at a rate of 3 m yr^−1^ during the period 2000–2019, with an average uncertainty of ±0.23 m yr^−1^ (95% confidence level). The elevation change uncertainties were estimated using a framework that accounts for heteroscedasticity (that is, variability in precision), which more reliably describes errors depending on terrain slope and the quality of stereo-correlation. The framework also accounts for spatial correlation of errors using multiple correlation ranges that better describe ASTER instrument noise^79^. These uncertainties were extensively validated using independent high-precision data from NASA’s Ice, Cloud and land Elevation Satellite (ICESat) and IceBridge missions, as well as LiDAR acquisitions^8^. Supplementary Table 6 reports statistics on spatial coverage, the temporal density of DEM data, thinning rates between 2000 and 2019, and associated uncertainties for each glacier dam.

To assess the impact of glacier thinning on GLOF magnitudes, we selected only *J* = 13 glacier lakes that burst out repeatedly (*n* > 5) between 2000 and 2019. For each dated outburst within a GLOF cycle, we obtained the closest temporal estimate of the cumulative elevation change of the glacier dam. We then fitted a hierarchical quantile regression model, that is, the same as in equations (1)–(13), to estimate the median of reported *V*_0_ and *Q*_p_ at a given glacier dam from the associated cumulative elevation change *h*. As we did when calculating temporal trends of *V*_0_, *Q*_p_ and *Z*, we chose an AL likelihood to estimate the conditional median of *V*_0_ and *Q*_p_, with the same (hyper-)prior distributions as in equations (7)–(12). We ran three parallel chains with 6,000 samples and 2,000 warm-up runs and found no numerical divergences. We report the posterior distributions of all model parameters in Supplementary Table 5.

## Online content

Any methods, additional references, Nature Portfolio reporting summaries, source data, extended data, supplementary information, acknowledgements, peer review information; details of author contributions and competing interests; and statements of data and code availability are available at 10.1038/s41586-022-05642-9.

## Supplementary information

Supplementary Tables This file contains Supplementary Tables 1–6.

## Data Availability

The GLOF inventory V2.0 is freely available at http://glofs.geoecology.uni-potsdam.de, and archived at 10.5281/zenodo.7326571. The ALOS DEM V3.2 is freely available at https://www.eorc.jaxa.jp/ALOS/en/dataset/aw3d30/aw3d30_e.htm. The RGI V6.0 is freely available at https://nsidc.org/data/nsidc-0770. Planet and RapidEye images are available under a basic license from the Education and Research programme from Planet Labs Inc. through the PlanetExplorer (https://www.planet.com/). Landsat images were obtained from the EarthExplorer (https://earthexplorer.usgs.gov/). Data on glacier elevation change for the period 2000–2019 are freely available at https://doi.org/10.6096/13.

## Code availability

All codes to reproduce the statistical analysis are available at https://github.com/geveh/IceDamFailures, and are archived at 10.5281/zenodo.7326865.
