# Supplementary material for: Less extreme and earlier outbursts of ice-dammed lakes since 1900
Source: Nature. 2023 Feb 15;614(7949):701–7. doi: 10.1038/s41586-022-05642-9 (PMC9946834; doi:10.1038/s41586-022-05642-9)
Supplement: Supplementary file 1 — This file contains Supplementary Tables 1–6. [file 41586_2022_5642_MOESM1_ESM.pdf]

nature portfolio

https://doi.org/10.1038/s41586-022-05642-9

Supplementary information

Less extreme and earlier outbursts of ice-dammed lakes since 1900

In the format provided by the authors and unedited

Supplementary Table 1: Prior and posterior distributions of the parameters in the regional models of V_0 with time t (Eqs. (1) to (13)).

**Period**	**Para-meter**	**Quantile** p	**Prior**	**Posterior Mean | 2.5% | 97.5% of HDI**
1900-2021	α	0.5	*Normal* (mean = 0, sd = 2.5)	0.12 | -0.73 | 0.93
1900-2021	β	0.5	*Normal* (mean = 0, sd = 2.5)	-0.12 | 0.22 | -0.04
1900-2021	σ_α	0.5	*Normal* (mean = 0, sd = 2.5) T(0,)	0.95 | 0.47 | 2.04
1900-2021	σ_β	0.5	*Normal* (mean = 0, sd = 2.5) T(0,)	0.07 | 0.00 | 0.23
1900-2021	κ	0.5	*Normal* (mean = 0, sd = 2.5) T(0,)	0.24 | 0.22 | 0.26
1900-2021	ς	0.5	*LkjCholesky*(1) on R	-0.34 | -0.98 | 0.71
1900-2021	α	0.9	*Normal* (mean = 0, sd = 2.5)	0.60 | -0.32 | 1.44
1900-2021	β	0.9	*Normal* (mean = 0, sd = 2.5)	-0.29 | -0.60 | 0.04
1900-2021	σ_α	0.9	*Normal* (mean = 0, sd = 2.5) T(0,)	1.01 | 0.50 | 2.09
1900-2021	σ_β	0.9	*Normal* (mean = 0, sd = 2.5) T(0,)	0.34 | 0.16 | 0.80
1900-2021	κ	0.9	*Normal* (mean = 0, sd = 2.5) T(0,)	0.08 | 0.07 | 0.09
1900-2021	ς	0.9	*LkjCholesky*(1) on R	-0.43 | -0.91 | 0.42
1990-2021	α	0.5	*Normal* (mean = 0, sd = 2.5)	0.15 | -0.86 | 1.14
1990-2021	β	0.5	*Normal* (mean = 0, sd = 2.5)	-0.27 | -0.77 | 0.28
1990-2021	σ_α	0.5	*Normal* (mean = 0, sd = 2.5) T(0,)	1.09 | 0.50 | 2.36
1990-2021	σ_β	0.5	*Normal* (mean = 0, sd = 2.5) T(0,)	0.46 | 0.05 | 1.31
1990-2021	κ	0.5	*Normal* (mean = 0, sd = 2.5) T(0,)	0.27 | 0.24 | 0.31
1990-2021	ς	0.5	*LkjCholesky*(1) on R	-0.27 | -0.92 | 0.73
1990-2021	α	0.9	*Normal* (mean = 0, sd = 2.5)	0.61 | -0.23 | 1.40
1990-2021	β	0.9	*Normal* (mean = 0, sd = 2.5)	-0.29 | -0.62 | 0.10
1990-2021	σ_α	0.9	*Normal* (mean = 0, sd = 2.5) T(0,)	0.94 | 0.45 | 1.96
1990-2021	σ_β	0.9	*Normal* (mean = 0, sd = 2.5) T(0,)	0.29 | 0.02 | 0.87
1990-2021	κ	0.9	*Normal* (mean = 0, sd = 2.5) T(0,)	0.08 | 0.07 | 0.09
1990-2021	ς	0.9	*LkjCholesky*(1) on R	-0.48 | -0.99 | 0.63

Notes: Priors refer to standardised input data pairs of $\log_{10}$-transformed V_0 and year for the period 1900-2021 using a mean of zero and unit standard deviation. T(·, ·) indicates a truncation of the distribution at an lower or upper boundary. sd, standard deviation. HDI, highest density interval.

Supplementary Table 2: Prior and posterior distributions of the parameters in the regional models of Q_p with time t (Eqs. (1) to (13)).

<table>
<tr><th>Period</th><th>Para-
meter</th><th>Quantile
p</th><th>Prior</th><th>Posterior
Mean | 2.5% | 97.5% of HDI</th></tr>
<tr><td>1900-2021</td><td>α</td><td>0.5</td><td>Normal (mean = 0, sd = 2.5)</td><td>-0.28 | -1.48 |0.93</td></tr>
<tr><td>1900-2021</td><td>β</td><td>0.5</td><td>Normal (mean = 0, sd = 2.5)</td><td>-0.11 | -0.32 |0.12</td></tr>
<tr><td>1900-2021</td><td>σ_α</td><td>0.5</td><td>Normal (mean = 0, sd = 2.5) T(0,)</td><td>1.38 | 0.70 |2.73</td></tr>
<tr><td>1900-2021</td><td>σ_β</td><td>0.5</td><td>Normal (mean = 0, sd = 2.5) T(0,)</td><td>0.22 | 0.07 |0.56</td></tr>
<tr><td>1900-2021</td><td>κ</td><td>0.5</td><td>Normal (mean = 0, sd = 2.5) T(0,)</td><td>0.25 | 0.23 |0.27</td></tr>
<tr><td>1900-2021</td><td>ς</td><td>0.5</td><td>LkjCholesky(1) on R</td><td>-0.22 | -0.86 | 0.63</td></tr>
<tr><td>1900-2021</td><td>α</td><td>0.9</td><td>Normal (mean = 0, sd = 2.5)</td><td>0.47 | -0.74 |1.63</td></tr>
<tr><td>1900-2021</td><td>β</td><td>0.9</td><td>Normal (mean = 0, sd = 2.5)</td><td>-0.32 | -0.46 | -0.15</td></tr>
<tr><td>1900-2021</td><td>σ_α</td><td>0.9</td><td>Normal (mean = 0, sd = 2.5) T(0,)</td><td>1.37 |0.69 |2.75</td></tr>
<tr><td>1900-2021</td><td>σ_β</td><td>0.9</td><td>Normal (mean = 0, sd = 2.5) T(0,)</td><td>0.14 | 0.02 |0.39</td></tr>
<tr><td>1900-2021</td><td>κ</td><td>0.9</td><td>Normal (mean = 0, sd = 2.5) T(0,)</td><td>0.11 | 0.10 |0.12</td></tr>
<tr><td>1900-2021</td><td>ς</td><td>0.9</td><td>LkjCholesky(1) on R</td><td>-0.37 | -0.94 | 0.53</td></tr>
<tr><td>1990-2021</td><td>α</td><td>0.5</td><td>Normal (mean = 0, sd = 2.5)</td><td>-0.09 | -1.52 |1.31</td></tr>
<tr><td>1990-2021</td><td>β</td><td>0.5</td><td>Normal (mean = 0, sd = 2.5)</td><td>-0.30 | -1.07 |0.51</td></tr>
<tr><td>1990-2021</td><td>σ_α</td><td>0.5</td><td>Normal (mean = 0, sd = 2.5) T(0,)</td><td>1.67 | 0.86 |3.25</td></tr>
<tr><td>1990-2021</td><td>σ_β</td><td>0.5</td><td>Normal (mean = 0, sd = 2.5) T(0,)</td><td>0.76 | 0.28 |1.80</td></tr>
<tr><td>1990-2021</td><td>κ</td><td>0.5</td><td>Normal (mean = 0, sd = 2.5) T(0,)</td><td>0.21 | 0.19 |0.24</td></tr>
<tr><td>1990-2021</td><td>ς</td><td>0.5</td><td>LkjCholesky(1) on R</td><td>-0.30 | -1.07 | 0.51</td></tr>
<tr><td>1990-2021</td><td>α</td><td>0.9</td><td>Normal (mean = 0, sd = 2.5)</td><td>0.53 | -0.91 |1.97</td></tr>
<tr><td>1990-2021</td><td>β</td><td>0.9</td><td>Normal (mean = 0, sd = 2.5)</td><td>-0.25 | -1.09 |0.64</td></tr>
<tr><td>1990-2021</td><td>σ_α</td><td>0.9</td><td>Normal (mean = 0, sd = 2.5) T(0,)</td><td>1.73 | 0.94 |3.14</td></tr>
<tr><td>1990-2021</td><td>σ_β</td><td>0.9</td><td>Normal (mean = 0, sd = 2.5) T(0,)</td><td>0.92 | 0.38 |2.02</td></tr>
<tr><td>1990-2021</td><td>κ</td><td>0.9</td><td>Normal (mean = 0, sd = 2.5) T(0,)</td><td>0.09 | 0.08 |0.10</td></tr>
<tr><td>1990-2021</td><td>ς</td><td>0.9</td><td>LkjCholesky(1) on R</td><td>-0.77 | -0.99 | -0.09</td></tr>
</table>

Notes: Priors refer to standardised input data pairs of $\log_{10}$-transformed Q_p and time for the period 1900-2021 using a mean of zero and unit standard deviation T(·, ·) indicates a truncation of the distribution at an lower or upper boundary. sd, standard deviation. HDI, highest density interval.

Supplementary Table 3: Prior and posterior distributions of the parameters in the regional models of Z with time t (Eqs. (1) to (13)).

Para-meter	Quantile p	Prior	Posterior Mean | 2.5% | 97.5% of HDI
α	0.5	*Normal* (mean = 0, sd = 2.5)	-0.06 | -1.14 | 1.02
β	0.5	*Normal* (mean = 0, sd = 2.5)	0.08 | -0.05 | 0.20
σ_α	0.5	*Normal* (mean = 0, sd = 2.5) T(0,)	1.29 | 0.65 | 2.65
σ_β	0.5	*Normal* (mean = 0, sd = 2.5) T(0,)	0.11 | 0.01 | 0.31
κ	0.5	*Normal* (mean = 0, sd = 2.5) T(0,)	0.16 | 0.14 | 0.19
ς	0.5	*LkjCholesky*(1) on R	-0.26 | -0.95 | 0.72

Notes: Priors refer to standardised input data pairs of *Z* and time for the period 1900-2021 using a mean of zero and unit standard deviation. T(·, ·) indicates a truncation of the distribution at an lower or upper boundary. sd, standard deviation. HDI, highest density interval.

Supplementary Table 4: Prior and posterior distributions of the parameters in the regional models of doy with time t (Eqs. (14) to (20)).

**Region**	**Parameter**	**Prior**	**Posterior Mean | 2.5% | 97.5% of HDI**
Andes	ζ	*Normal* (mean = 0, sd = 2.5)	-4.59 | -7.27 | -2.70
Andes	η	*Normal* (mean = 0, sd = 0.75)	-0.01 | -1.16 | 1.18
Andes	φ	*Gamma* (shape = 2, rate = 0.01)	1.36 | 0.83 | 1.94
European Alps	ζ	*Normal* (mean = 0, sd = 2.5)	0.16 | 0.10 | 0.22
European Alps	η	*Normal* (mean = 0, sd = 0.75)	-0.15 | -0.21 | -0.09
European Alps	φ	*Gamma* (shape = 2, rate = 0.01)	3.78 | 2.90 | 4.78
NW North America	ζ	*Normal* (mean = 0, sd = 2.5)	0.37 | 0.33 | 0.42
NW North America	η	*Normal* (mean = 0, sd = 0.75)	-0.12 | -0.18 | -0.07
NW North America	φ	*Gamma* (shape = 2, rate = 0.01)	2.35 | 2.10 | 2.63
High Mountain Asia	ζ	*Normal* (mean = 0, sd = 2.5)	0.27 | 0.21 | 0.33
High Mountain Asia	η	*Normal* (mean = 0, sd = 0.75)	-0.18 | -0.25 | -0.11
High Mountain Asia	φ	*Gamma* (shape = 2, rate = 0.01)	2.65 | 2.16 | 3.18
Scandinavia	ζ	*Normal* (mean = 0, sd = 2.5)	0.33 | 0.23 | 0.42
Scandinavia	η	*Normal* (mean = 0, sd = 0.75)	-0.05 | -0.12 | 0.03
Scandinavia	φ	*Gamma* (shape = 2, rate = 0.01)	2.25 | 1.72 | 2.86
Iceland	ζ	*Normal* (mean = 0, sd = 2.5)	0.25 | 0.17 | 0.33
Iceland	η	*Normal* (mean = 0, sd = 0.75)	0.08 | -0.03 | 0.19
Iceland	φ	*Gamma* (shape = 2, rate = 0.01)	2.08 | 1.64 | 2.57

Notes: Priors refer to input data pairs of *doy*, rescaled to $y_i \in (-\pi, \pi)$, and time, standardised using a mean of zero and unit standard deviation, for the period 1900-2021. sd, standard deviation. HDI, highest density interval.

Supplementary Table 5: Prior and posterior distributions of the parameters in the local models of V_0 and Q_p with cumulative glacier elevation change h (Eqs. (1) to (13)).

**Response**	**Para-meter**	**Quantile p**	**Prior**	**Posterior Mean | 2.5% | 97.5% of HDI**
V_0	α	0.5	*Normal* (mean = 0, sd = 2.5)	-0.08 | -0.91 | 0.73
V_0	β	0.5	*Normal* (mean = 0, sd = 2.5)	0.05 | -0.09 | 0.21
V_0	σ_α	0.5	*Normal* (mean = 0, sd = 2.5) T(0,)	1.18 | 0.69 | 2.07
V_0	σ_β	0.5	*Normal* (mean = 0, sd = 2.5) T(0,)	0.13 | 0.01 | 0.34
V_0	κ	0.5	*Normal* (mean = 0, sd = 2.5) T(0,)	0.12 | 0.10 | 0.15
V_0	ς	0.5	*LkjCholesky*(1) on R	0.40 | -0.72 | 0.98
Q_p	α	0.5	*Normal* (mean = 0, sd = 2.5)	-0.18 | -0.74 | 0.39
Q_p	β	0.5	*Normal* (mean = 0, sd = 2.5)	0.10 | -0.07 | 0.24
Q_p	σ_α	0.5	*Normal* (mean = 0, sd = 2.5) T(0,)	0.93 | 0.59 | 1.50
Q_p	σ_β	0.5	*Normal* (mean = 0, sd = 2.5) T(0,)	0.16 | 0.02 | 0.41
Q_p	κ	0.5	*Normal* (mean = 0, sd = 2.5) T(0,)	0.17 | 0.14 | 0.20
Q_p	ς	0.5	*LkjCholesky*(1) on R	0.54 | -0.39 | 0.98

Notes: Priors refer to standardised input data pairs of Z and time for the period 2000-2019 using a mean of zero and unit standard deviation. T(·, ·) indicates a truncation of the distribution at an lower or upper boundary. sd, standard deviation. HDI, highest density interval.

Supplementary Table 6: Summary statistics for each glacier dam, for which we obtained glacier elevation change h between 2000 and 2019.

RGI ID	Area (km²)	Mean elevation change (m yr^{-1})	Error (2σ) in mean elevation change (m yr^{-1})	Area measured (%)	Average valid observations in 20 years	Covered by ArcticDEM
RGI60-01.00709	7.19	-3.57	0.32	98.5	21.9	Yes
RGI60-01.01521	18.65	-4.95	0.26	95.2	17.0	Yes
RGI60-01.06618	9.72	-4.61	0.25	98.8	31.0	Yes
RGI60-01.09061	19.81	-1.93	0.23	99.8	30.1	Yes
RGI60-01.15645	53.38	-1.64	0.27	99.4	30.0	Yes
RGI60-01.17774	28.64	-1.33	0.25	97.5	15.7	Yes
RGI60-01.21005	9.33	-1.75	0.3	99.7	12.8	Yes
RGI60-01.23641	51.27	-2.06	0.27	96.7	23.1	Yes
RGI60-08.02384	2.53	-1.38	0.3	100.0	33.9	Yes
RGI60-11.02072	8.14	-1.51	0.29	99.9	55.7	No
RGI60-11.02822	6.12	-3.93	0.33	95.1	59.3	No
RGI60-13.05000	34.23	-1.33	0.23	99.7	29.5	No
RGI60-14.07098	3.74	-0.66	0.41	97.4	32.0	No
RGI60-17.15859	20.89	-4.22	0.28	96.9	39.0	No
